# Comprehensive analysis of RNA-seq and whole genome sequencing data reveals no evidence for SARS-CoV-2 integrating into host genome

**DOI:** 10.1007/s13238-021-00861-8

**Published:** 2021-08-02

**Authors:** Yu-Sheng Chen, Shuaiyao Lu, Bing Zhang, Tingfu Du, Wen-Jie Li, Meng Lei, Yanan Zhou, Yong Zhang, Penghui Liu, Yong-Qiao Sun, Yong-Liang Zhao, Ying Yang, Xiaozhong Peng, Yun-Gui Yang

**Affiliations:** 1 CAS Key Laboratory of Genomic and Precision Medicine, Collaborative Innovation Center of Genetics and Development, College of Future Technology, Beijing Institute of Genomics, Chinese Academy of Sciences, Beijing, 100101 China; 2 China National Center for Bioinformation, Beijing, 100101 China; 3 National Kunming High-level Biosafety Primate Research Center, Institute of Medical Biology, Chinese Academy of Medical Sciences and Peking Union Medical College, Kunming, 650031 China; 4 Institute of Stem Cell and Regeneration, Chinese Academy of Sciences, Beijing, 100101 China; 5 University of Chinese Academy of Sciences, Beijing, 100049 China; 6 State Key Laboratory of Medical Molecular Biology, Department of Molecular Biology and Biochemistry, Institute of Basic Medical Sciences, Medical Primate Research Center, Neuroscience Center, Chinese Academy of Medical Sciences, School of Basic Medicine Peking, Union Medical College, Beijing, 100005 China

**Dear Editor,**

Severe acute respiratory syndrome coronavirus 2 (SARS-CoV-2) is an RNA virus of the Coronaviridae family causing the outbreak and worldwide pandemic of coronavirus disease 2019 (COVID-19) (Zhou et al., 2020b; Zhu et al., 2020b). As an emergent and unprecedented global threat to public health, it has affected about 162 million individuals with over 3.3 million deaths until the middle of May. In attributed to the extensive studies on SARS-CoV-2, several kinds of vaccines are currently available which brings hope to human society for alleviating and eventually preventing COVID-19 epidemic (Zhu et al., 2020a; Xia et al., 2021). However, a better understanding of viral pathogenesis, particularly the viral-host interaction, are needed to develop effective interventions.

Viruses are mainly separated into four groups based on the types of genome they belong to, including dsDNA, ssDNA, dsRNA and ssRNA (Baltimore, 1971). All viruses need host cells for their reproduction through introducing their genetic materials into the infected cells and hijacking certain cellular machinery, such as ribosome, polymerase and so on, to produce viral particles and related functional proteins, like replicative enzymes, the antagonists of host immune response, etc. Noticeable, some RNA viruses are capable of inserting their reverse-transcribed DNA into host genome and then produce viral RNA through host-dependent transcription pathway. To fulfill this, two mechanisms have been reported: (1) some retroviruses, such as HIV (human immunodeficiency virus), ASV (avian sarcoma virus) and PFV (prototype foamy virus), can encode their own reverse transcriptase to synthesize a complementary ssDNA for the host genome integration (Sultana et al., 2017; Grandgenett and Aihara, 2018); (2) for some non-retroviruses, such as LCMV (lymphocytic choriomeningitis virus) and BDV (borna disease virus), they have been reported to utilize elements of endogenic transposons, like endogenous retroviral transposon IAP (intracisternal A-particle) or LINE1 (long interspersed nuclear element 1), for recombination with host genome (Horie and Tomonaga, 2011). It should be noted that the endogenous retrotransposons are inactivated in mammalian cells except for early developmental stage or pathological conditions, such as tumors (Geis and Goff, 2020).

As a positive single-strand RNA virus, SARS-CoV-2 generates sub-genomic RNAs through a mechanism termed discontinuous extension of minus strands, and further synthesizes its proteins in cytoplasm of infected cells (V’Kovski et al., 2021). However, one recent study revealed that SARS-CoV-2 infection induced retrotransposon activation, leading to the formation of chimeric virus-retrotransposon RNA (Yin et al., 2021). Zhang et al. also observed the host genome integration of SARS-CoV-2 under overexpressing LINE1 in cultured human cells (Zhang et al., 2021). These reports indicate a likely chance of SARS-CoV-2 integration into host genome (Yin et al., 2021; Zhang et al., 2021), which have fueled concerns about its potential long-term health threat in the infected individuals. Thus, more evidences are explicitly required to address this issue.

To investigate whether or not the SARS-CoV-2 is capable of integrating into host genome, we performed RNA-seq for SARS-CoV-2 infected 293T, Huh-7 and Calu-3 cells and validated the chimeric reads from both SARS-CoV-2 and human genomes. Firstly, we assessed the reproducibility of chimeric junctions in one sample or amongst three samples. CPM (counts per million) was estimated by the counts of all chimeric reads along gene body and normalized by sequencing depth (Fig. S1A). After filtering genes with low chimeric levels, there were 126 chimeric genes identified in Huh-7 and Calu-3 cells, while none was detected in 293T cells. Moreover, 18 of them were overlapped between these two cell lines, and covered various RNA types, including mRNA, lncRNA and miscellaneous RNA (misc RNA) (Fig. S1B). We further checked the proportion of reads derived from SARS-CoV-2 in each cell line and found that only 0.4% reads were aligned to viral genome in 293T cells, much less than that of the other two cell lines (Fig. S1C), which is supported by read counts for all genes containing chimeric reads (Fig. S1D). As such, we used the viral depth, instead of sequencing depth, for CPM normalization.

Using the normalized CPM, there were 347, 3,107 and 4,171 chimeric genes identified from 293T, Huh-7 and Calu-3 cells, respectively (Figs. 1A and S1E). Most of them were the fusion of viral RNA with mRNA from host cells (Fig. S1E). We further obtained 132 conserved chimeric genes from all of three cell lines (Fig. 1B) and analyzed the categories of RNA types. The results showed that the chimeric genes were preferentially mRNA (Fig. 1C), likely due to the abundant expression of mRNA relative to other RNA types. We then displayed the chimeric events for 5 randomly picked conserved chimeric genes (*RPL3*, *GPI*, *ATP5F1A*, *EEF2* and *RPS19*) among 293T, Huh-7 and Calu-3 cell lines, and found that the chimeric events were extremely diverse in these cells (Figs. 1D and S1F). Moreover, we analyzed the reads number of each chimeric event along chimeric gene, and found that most of them could be identified only once in each cell line (Fig. S1G). Collectively, these results suggest that RNA chimeric event seems to be a random case for an individual infected cell.

**Figure 1:**
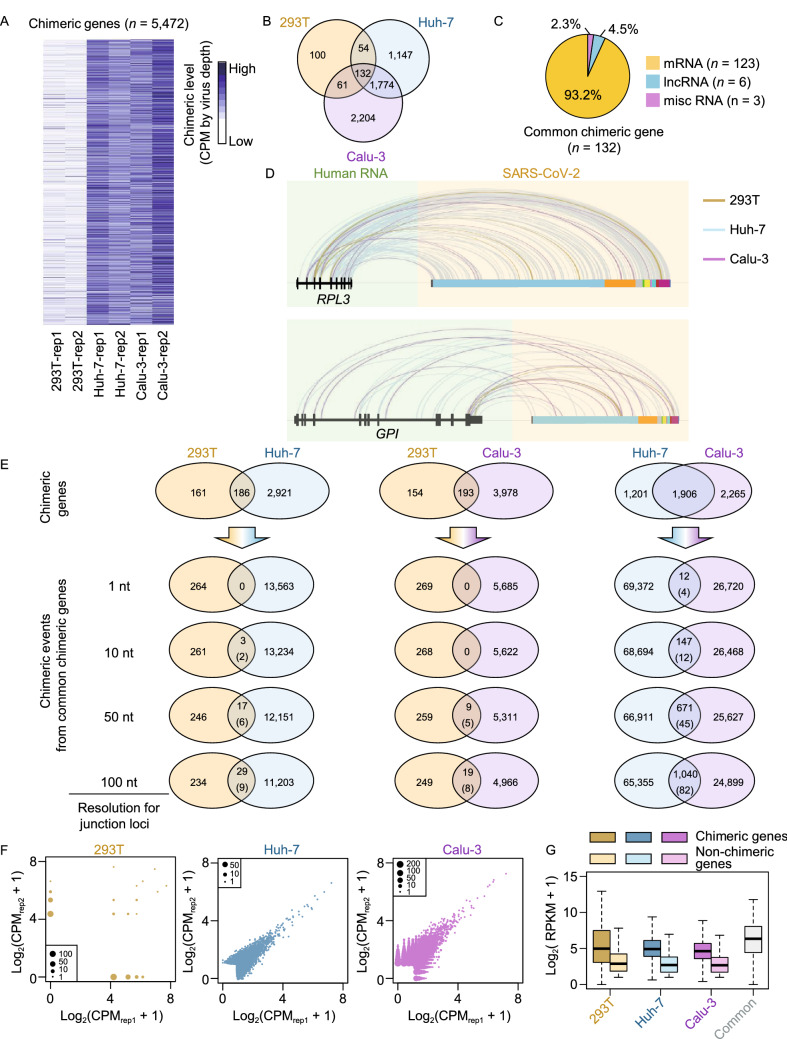
**Chimeric genes of human and SARS-CoV-2 were frequently detected among highly expressed gene by RNA-seq**. (A) Heatmap displaying the chimeric level for filtered chimeric genes in each sample. The chimeric level was defined as CPM of chimeric reads and normalized by depth of reads from SARS-CoV-2. (B) Venn diagram showing the number of shared and specific chimeric genes among 3 human cell lines. (C) Pie chart displaying proportion of RNA types for 132 common chimeric genes. (D) Integrative genomics viewer (IGV) tracks displaying the junction loci in both human RNA and SARS-CoV-2 RNA for common chimeric genes *RPL3* (upper) and *GPI* (lower). The lines with different colors indicate the sources of chimeric reads, where yellow, blue and pink represents 293T, Huh-7 and Calu-3 cells, respectively. Blocks with different colors represent 5’UTR, ORF1ab, S, ORF3a, E, M, ORF6, ORF7ab, ORF8, N and 3’UTR along the SARS-CoV-2 genome. (E) Venn diagrams showing the number of shared chimeric genes for each two cell lines (top) and conserved chimeric events on shared chimeric genes at different resolutions (1 nt, 10 nt, 50 nt and 100 nt). For venn diagrams of chimeric events, the value in parenthesis represents the number of chimeric genes containing the shared chimeric events. (F) Scatter plot displaying the chimeric levels for chimeric genes in two replicates. The sizes of scatters represent number of chimeric genes with the same distribution of chimeric levels in two replicates. (G) Boxplot displaying the distributions of expression level for chimeric genes (dark color) and non-chimeric genes (light color) in each cell line. The grey block represents expression levels of 132 common chimeric genes

To further investigate whether chimeric events were species-specific, we performed RNA-seq on SARS-CoV-2 infected green monkey Cos-7 and rhesus monkey MA-104 cells. Intriguingly, compared to Cos-7, there was a large amount of viral RNA detected in MA-104 sample (Fig. S1H). Moreover, most chimeric genes estimated by CPM contained only one read and belonged to mRNA (Fig. S1I and S1J). Similar to human cell line, the chimeric events could only be found once in each monkey cell line (Fig. S1K). Thus, low incidence of chimeric event was observed in the infected cells from human and other species.

We next tried to determine whether the diversity of chimeric events among different cell types results from random integration or integration preference for specific cell types, we displayed the overlapped chimeric genes and events for each two cell lines (Fig. 1E). Though over half chimeric genes overlapped in both cell lines, the shared chimeric events were extremely low. We then used different resolutions to define the chimeric events to determine if the virus-host integration might be enriched in hot-spot regions instead of specific nucleotides, and found these events were still not conserved (Fig. 1E). Given that the low reproducibility might be contributed by heterogeneity from different cell lines, we further analyzed the number of overlapped chimeric genes and events between two sequencing replicates from one cell line (Fig. S2A). Unexpectedly, the chimeric genes in 293T cells were diverse with only few chimeric events identified in both replicates (Fig. S2A and S2B), which might be owing to the much less SARS-CoV-2 reads in this cell line.

Then we compared the chimeric level for each chimeric gene in two replicates (Fig. 1F). The relatively high similarity between two replicates was found in both Huh-7 and Calu-3 cells, suggesting that SARS-CoV-2 might select genes to fuse by some unknow elements in individual cells while the loci seemed random. To further search for the key elements, we compared the expression levels between chimeric and non-chimeric genes, and observed that chimeric genes had a remarkably higher expression level than those of non-chimeric genes (Figs. 1G and S3A). After evaluating the proportions of chimeric genes with low to high expression level, we also found that chimeric events tended to occur in highly expressed host genes (Fig. S3B). In addition, the chimeric loci along virus were also enriched in N region, the most abundant sub-genomic RNA of SARS-CoV-2 (Fig. S3C and S3D), indicating that chimeric events were positively correlated with expression levels of both host and virus RNAs.

We next tested the conservativeness of the preference of chimeric events for highly expressed genes in monkey cell lines. The reproducibility for two replicates were first validated for chimeric genes (Fig. S4A) and the chimeric events for the shared chimeric genes were then analyzed (Fig. S4B and S4C). Similar to the findings in human cells, reproducibility of chimeric events in overlapped genes were extremely low between two replicates, suggesting that the integrating events were most likely randomly occurred. In addition, chimeric genes showed preferred enrichment in high expression genes in both monkey cell lines (Fig. S4D–F). Moreover, the viral chimeric loci were highly correlated to the expression of viral sub-genomic RNA in MA-104 (Fig. S4G and S4H), which was also observed in human cell lines.

Recently, Zhang et al. reported that SARS-CoV-2 RNA might integrate into host genome *via* reverse-transcription, and overexpressed LINE1 might stimulate the reverse-transcribed SARS-CoV-2 integration into host genome (Zhang et al., 2021), which prompted us to determine whether or not the correlation of chimeric events with high expression of host genes was attributed to the viral integration into the locations of DNA elements, such as promoters and enhancers. We first analyzed the number of chimeric genes in each chromosome, and didn’t identify any evidence of preferred viral integration into some chromosomes (Fig. S5A and S5B).

We then estimated the accumulated chimeric and expression levels of 100 genes in each of 617 bins obtained by sequentially cutting the genome along chromosomes (each containing 100 genes). Intriguingly, though there was no preference for whole chromosomes, the bins represented positive correlation between expression and chimeric levels (Fig. 2A), which might be explained by possible viral integration into transcription regular elements along host DNA. Additionally, we found that the bin in chromosome 14 (chr14: 39,385,404-49,852,821) had both the highest chimeric level and gene expression, and was well conserved among 3 cell lines (Figs. 2A and S5C). However, this much enhanced chimeric level was likely to be contributed by one single gene, the non-coding *RN7SL1*, with the highest expression. Next, we tried to use the frequency of chimeric genes to explain whether the chimeric preference to highly expressed genes resulted from integration (Fig. S5D and S5E). However, no evidence could support this hypothesis.

**Figure 2:**
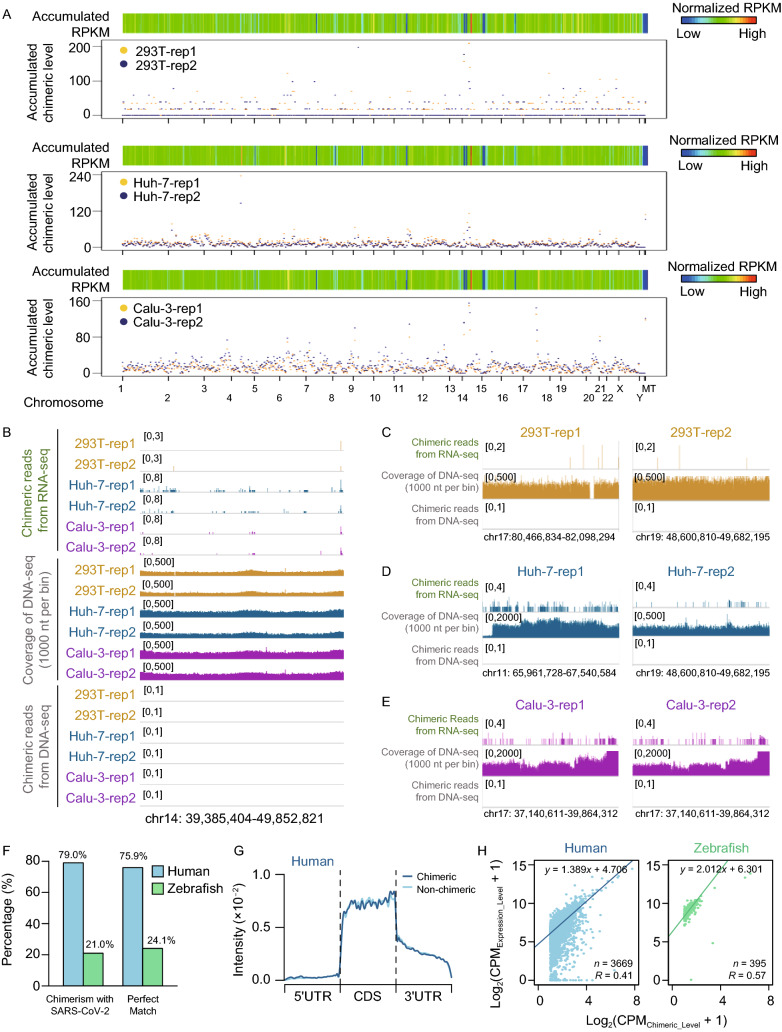
**Whole genome sequencing and mixed RNA-seq library reveal chimeric reads are falsely generated during library construction but not integration of SARS-CoV-2 into host genome**. (A) Heatmap showed the accumulated expression score of each bin along human genome, and scatter plot below displayed the accumulated chimeric levels of corresponding bins for each cell line. (B) IGV tracks displaying the expression level (top), coverage of whole genome sequencing (middle) and genomic insertion signal (bottom) along chr14: 39,385,404-49,852,821. No viral integrating signal could be detected among all samples. (C-E) IGV tracks displaying the expression level (top), coverage of whole genome sequencing (middle) and genomic insertion signal (bottom) along bins in 293T (C), Huh-7 (D) and Calu-3 (E). (F) Proportions of viral chimeric reads with human (blue) and zebrafish RNA (green) were shown in left, while the corresponding ratio of sequencing depth for human and zebrafish, which defined as number of perfect matched reads, were shown in right. (G) Distribution of chimeric (dark blue) and non-chimeric reads (light blue) across the length of chimeric human mRNAs. 5’UTRs, CDSs, and 3’UTRs of human mRNAs were individually binned into regions spanning 1% of their total length, and the percentages of chimeric and non-chimeric reads that fall within each bin were determined, respectively. (H) Scatter plot displaying the correlation between chimeric level and counts of perfect matched fragments (CPM) to chimeric genes for human (left) and zebrafish (right) from the same mixed library

To determine whether or not the viral integration directly correlate with the high gene expression level, we further performed whole genome sequencing in parallel with RNA-seq for the SARS-CoV-2 infected samples (Fig. S5F). The sequencing coverage was about 30× and more than 95% chimeric events were covered at least 10×, however, no chimeric junction reads between human and SARS-CoV-2 could be found in bins in terms of the highest chimeric level (Fig. 2B) and the most frequency of chimeric genes (Fig. 2C–E). Moreover, there was no reads aligned to SARS-CoV-2 reference genome in all genome sequencing data, indicating that the viral integration into host DNA fragments is most unlikely through reverse-transcription.

Since the chimeric events truly existed in RNA-seq and showed preference to highly expressed genes, we speculate that they were likely caused by random priming between RNA templates in synthesizing first-strand and second-strand cDNA. However, we noticed that the chimeric loci showed random distributions, instead of preference to 5’ or 3’ termini, along both viral genome and host genes (Figs. 1D, S1F and S2B). It seemed that the original viral and host RNAs for chimeric genes were first cleaved into fragments which then formed the chimeric events between SARS-CoV-2 and host. In such a case, it is important to clarify sources of the chimeric events from either the digested RNA fragments or fragmentation during library construction.

To verify this speculation, we constructed the sequencing library with mixed RNA samples from SARS-CoV-2 infected Huh-7 cells and normal zebrafish embryos (Huh-7 RNA : Zebrafish RNA = 7 : 3), and then performed RNA-seq for this mixed library. With the same analysis pipelines, we found that the observed proportion of reads aligned to different species were similar as expected (Fig. S6A). Intriguingly, the chimeric events were not only identified between SARS-CoV-2 and Huh-7 but also between SARS-CoV-2 and zebrafish, although most chimeric events were contributed by only one read (Fig. S6B). Moreover, we consistently observed that both of viral chimeric genes in Huh-7 and zebrafish showed preference to the highly expressed genes (Fig. S6C–E), and the ratios of viral chimeric reads in human versus zebrafish was proportionally correlated to the ratio of read number of human to zebrafish (Fig. 2F). We further calculated the distributions of chimeric and non-chimeric reads along chimeric genes, respectively, and observed that they exhibited similar distributive patterns in both chimeric human and zebrafish genes (Fig. 2G and S6F). In addition, for viral loci, chimeric reads for both human and zebrafish also displayed same distribution and were enriched in N sub-genomic RNA (Fig. S6G). All these results indicate that the chimeric events of SARS-CoV-2 and host cells from RNA-seq were false-positive and mainly emerged during library constructions. We then performed regression analysis for chimeric level with gene expression levels using CPM or RPKM (reads per kilobase per million mapped reads, normalizing gene expression with gene length) (Figs. 2H and S6H). Although both methods produced positive correlations, the regression lines by gene CPM for human and zebrafish were more similar than those by RPKM (Fig. 2H), suggesting the chimeric level were mainly related to the fragments in library. Additionally, we also found some reads representing the chimeric events between human and zebrafish (Fig. S2I), further supporting that the chimeric events artificially occur during library construction.

SARS-CoV-2 is highly contagious, and can cause severe clinical symptoms through infringing multi-organ systems (Zhou et al., 2020a). Therefore, it is necessary to clarify whether or not the viral RNA sequence has the potential to be integrated into the host genome imposing a long-term health risk. The recent report revealed the integrating events of SARS-CoV-2 with host genome in LINE1 overexpressing human cells (Zhang et al., 2021). To further clear this issue, we performed both RNA-seq and whole genome sequencing on SARS-CoV-2 infected human and monkey cells. Although we identified the presence of chimeric reads in RNA-seq, the analysis on bins with highest chimeric level and the most frequency of chimeric genes, in corroboration with the whole genome sequencing data, failed to show any chimeric reads (Fig. 2B–E). More importantly, chimeric reads were also identified in uninfected zebrafish embryos when mixing their RNAs with the RNAs from infected human cells at library construction step, therefore, providing the solid evidence that chimeric reads in viral infected samples are artificially introduced mainly through random ligations during library construction. In support, another team also observed the similar results based on their RNA-seq analysis (Yan et al., 2021). Although Zhang et al. found that SARS-CoV-2 could be reverse-transcribed and integrated into the genome of cultured cells overexpressing LINE1 (Zhang et al., 2021), it might be mainly due to the activation of retrotransposon but not natural characters of SARS-CoV-2. However, using the same long-read sequencing system, Smits et al. didn’t identify any LINE1 mediated SARS-CoV-2 genomic integration through analyzing genomic DNA from SARS-CoV-2 infected HEK293T cells without LINE1 overexpression (Smits et al., 2021). In support, our findings provide clear evidence that SARS-CoV-2 does not integrate into host genome, which will certainly help to alleviate the public concern about this issue.

## Supplementary Information

Below is the link to the electronic supplementary material.

Supplementary file 1 (PDF 2006 kb)

Supplementary file 2 (XLSX 15 kb)

Supplementary file 3 (XLSX 9484 kb)

Supplementary file 4 (XLSX 3099 kb)

Supplementary file 5 (XLSX 3677 kb)

Supplementary file 6 (XLSX 843 kb)

Supplementary file 7 (XLSX 6450 kb)
