# Supplementary material for: Comprehensive analysis of RNA-seq and whole genome sequencing data reveals no evidence for SARS-CoV-2 integrating into host genome
Source: Protein Cell. 2021 Aug 2;13(5):379–85. doi: 10.1007/s13238-021-00861-8 (PMC8327898; doi:10.1007/s13238-021-00861-8)
Supplement: Supplementary file 1 — (PDF 2006 kb) [file 13238_2021_861_MOESM1_ESM.pdf]

METHODS

Cell culture and virus infection

HEK293T (Cell Resource Center, Institute of Basic Medicine Chinese Academy of Medical Sciences, 3111C0001CCC000091), Huh-7 (Cell Resource Center, Institute of Basic Medicine Chinese Academy of Medical Sciences, 3111C0001CCC000679), Calu-3 (Cell Resource Center, Institute of Basic Medicine Chinese Academy of Medical Sciences, 3111C0001CCC000032), Cos-7 (Kunming Cell Bank, Chinese Academy of Sciences, 3153C0001000000037) and MA-104 (Chinese Typical Culture Preservation Center Cell Bank, 3142C0001000000041) cells were cultured in DMEM (ThermoFisher,0030034DJ) supplemented with 10% fetal bovine serum (Gibco, 10099-141C) and penicillin-streptomycin (ThermoFisher,15140122) at 37℃, 5% CO2.

Cells were infected with SARS-CoV-2 (CDC of Guangdong province, GD108#), at a multiplicity of infection (MOI) of 0.1, and were collected for RNA isolation 24 hours post virus infection. All experiments with the SARS-CoV-2 virus were performed in the BSL-4 laboratory.

RNA purification, library construction and sequencing

Total RNA from SARS-CoV-2 infected cell samples was extracted with Trizol reagent (Invitrogen, 15596026), and then subjected to the rRNA depletion with the Ribo-off rRNA Depletion Kit (Human/Mouse/Rat)(Vazyme, N406-02) following the manufacturer's instructions. Libraries were constructed using the KAPA RNA HyperPrep Kit (KAPA Biosystems, KK8541) following the manufacturer's instructions. Sequencing was performed on Illumina NovaSeq 6000 system with paired end 150 bp read length. Meanwhile, mixed sample of SARS-CoV-2 infected Huh-7 cells with zebrafish embryonic RNA was also used to prepare RNA-seq libraries, serving as the artificial chimeric RNA-seq reads control. For each infected sample, two replicates were performed, while three replicates were performed for the mixed sample.

Genomic DNA isolation, library construction and whole genome sequencing

DNA samples were isolated by Universal Genomic DNA Kit (CWBIO, CW2298) according to the manufacturer's instructions. The optical density values at 260/280 were approximately 1.6~1.8. Genomic DNA of the same SARS-CoV-2 infected cell lines was prepared and whole genome shotgun libraries were constructed by using

TruePrep DNA library Prep kit V2 for Illumina (Vazyme, Cat No. TD501-TD503), followed by sequencing on Illumina NovaSeq 6000 with paired end 150 bp read length. For each infected sample, two replicates were performed. About 100 Gb data were obtained for each replicate (Table S1).

Bioinformatics analysis of RNA-seq data

Raw FASTQ reads was processed to filter low quality bases and cut adapter sequence by fastp (version 0.20.1)(Chen et al., 2018). The clean RNA-seq reads from SARS-CoV-2 infected cell were aligned to host genome appending with SARS-CoV-2 genome (NC_045512) by using STAR (version 2.7.7a, parameters as followed: --chimOutType Junctions SeparateSAMold WithinBAM HardClip --chimSegmentMin 50 --chimScoreJunctionNonGTAG 0 --alignSJstitchMismatchNmax -1 -1 -1 -1 --chimJunctionOverhangMin 50 --outSAMtype BAM SortedByCoordinate --quantMode TranscriptomeSAM GeneCounts)(Dobin et al., 2013). The versions of genomes include GRCh38 for human (annotation release 102), Vero_WHO_p1.0 for green monkey (NCBI Chlorocebus sabaeus annotation release 102) and Mmul_10 for rhesus monkey (NCBI Macaca mulatta annotation release 103), and GRCz11 for zebrafish (NCBI: GCA_000002035.4). The duplicates were discarded. For RNA-seq reads from the mixed libraries of SARS-CoV-2 infected cells and uninfected zebrafish embryos were also aligned to human genome, zebrafish genome and SARS-CoV-2 genome with the same pipeline. The chimeric reads were also gained between each two genomes.

Bioinformatics analysis of whole genome sequencing data

Reads from whole genome sequencing data were mapped to human genome plus SARS-CoV-2 genome by using BWA (version 0.7.17)(Li and Durbin, 2009), and genome coverage statistics was obtained by samtools (version 1.12)(Li et al., 2009).

Gene expression analysis

Gene expression level were determined by RPKM (Reads Per Kilobase per Million mapped reads) and reads counts of genes were calculated by cufflinks (Trapnell et al., 2012). The expressed genes were then filtered by RPKM ≥ 1. To note, in Fig. 4c, the expression levels were defined as CPM without normalization of transcript length to represent the fragment depth of each transcript, directly. The p value for difference of

expressions between chimeric genes and non-chimeric genes were calculated by Mann-Whitney U test (Table S2, S4 and S6).

Chimeric gene analysis

Chimeric reads between host and SARS-CoV-2 were obtained via the above STAR analysis pipeline. For these reads, the fragments from host genome were annotated by corresponding genome annotation reference by bedtools intersectBed (Quinlan and Hall, 2010). The chimeric level was then defined as CPM (Counts Per Million reads), which were calculated by all chimeric reads from same gene and normalized by viral reads depth but not sequencing depth, except for results in Extended Data Fig. 1. The chimeric genes were then filtered by CPM ≥ 1 (Table S3, S5 and S6).

The chimeric events were defined by loci of junctions between host and SARS-CoV-2. Chimeric events at different resolutions for (10 nt, 50 nt and 100 nt) were re-defined by corresponding steps, which were obtained by sliding windows at various resolution along host and viral genome.

Analysis for accumulated expression and chimeric levels

The accumulated expression and chimeric levels were defined by sum of genes in related bins. Genes were first sorted in order of loci along each chromosome and then separated into one bin for each 100 genes. Those genes in the terminal of chromosome were defined as one bin though the number of genes might be less than 100. The accumulated expression level and chimeric level of each bin were calculated by sums of expression level and chimeric level from genes in the bin, respectively.

Statistics and reproducibility

Non-parametric Mann-Whitney U-test (Wilcoxon rank-sum test, two-sided) is applied for calculating p value to assess the statistical significance of differences between two groups, which has also been mentioned in the related figure legends. Linear regression model is performed to evaluate the correlation between chimeric level and expression level (both RPKM and CPM).

Data Availability

The RNA-seq and whole genome sequencing data supporting the conclusions of this

article has been deposited in the Genome Sequence Archive under accession number CRA004187 linked to the project PRJCA004871.

REFERENCES

Chen, S., Zhou, Y., Chen, Y., and Gu, J. (2018). fastp: an ultra-fast all-in-one FASTQ preprocessor. Bioinformatics *34*, i884-i890.

Dobin, A., Davis, C.A., Schlesinger, F., Drenkow, J., Zaleski, C., Jha, S., Batut, P., Chaisson, M., and Gingeras, T.R. (2013). STAR: ultrafast universal RNA-seq aligner. Bioinformatics *29*, 15-21.

Li, H., and Durbin, R. (2009). Fast and accurate short read alignment with Burrows-Wheeler transform. Bioinformatics *25*, 1754-1760.

Li, H., Handsaker, B., Wysoker, A., Fennell, T., Ruan, J., Homer, N., Marth, G., Abecasis, G., Durbin, R., and Genome Project Data Processing, S. (2009). The Sequence Alignment/Map format and SAMtools. Bioinformatics *25*, 2078-2079.

Quinlan, A.R., and Hall, I.M. (2010). BEDTools: a flexible suite of utilities for comparing genomic features. Bioinformatics *26*, 841-842.

Trapnell, C., Roberts, A., Goff, L., Pertea, G., Kim, D., Kelley, D.R., Pimentel, H., Salzberg, S.L., Rinn, J.L., and Pachter, L. (2012). Differential gene and transcript expression analysis of RNA-seq experiments with TopHat and Cufflinks. Nat Protoc *7*, 562-578.

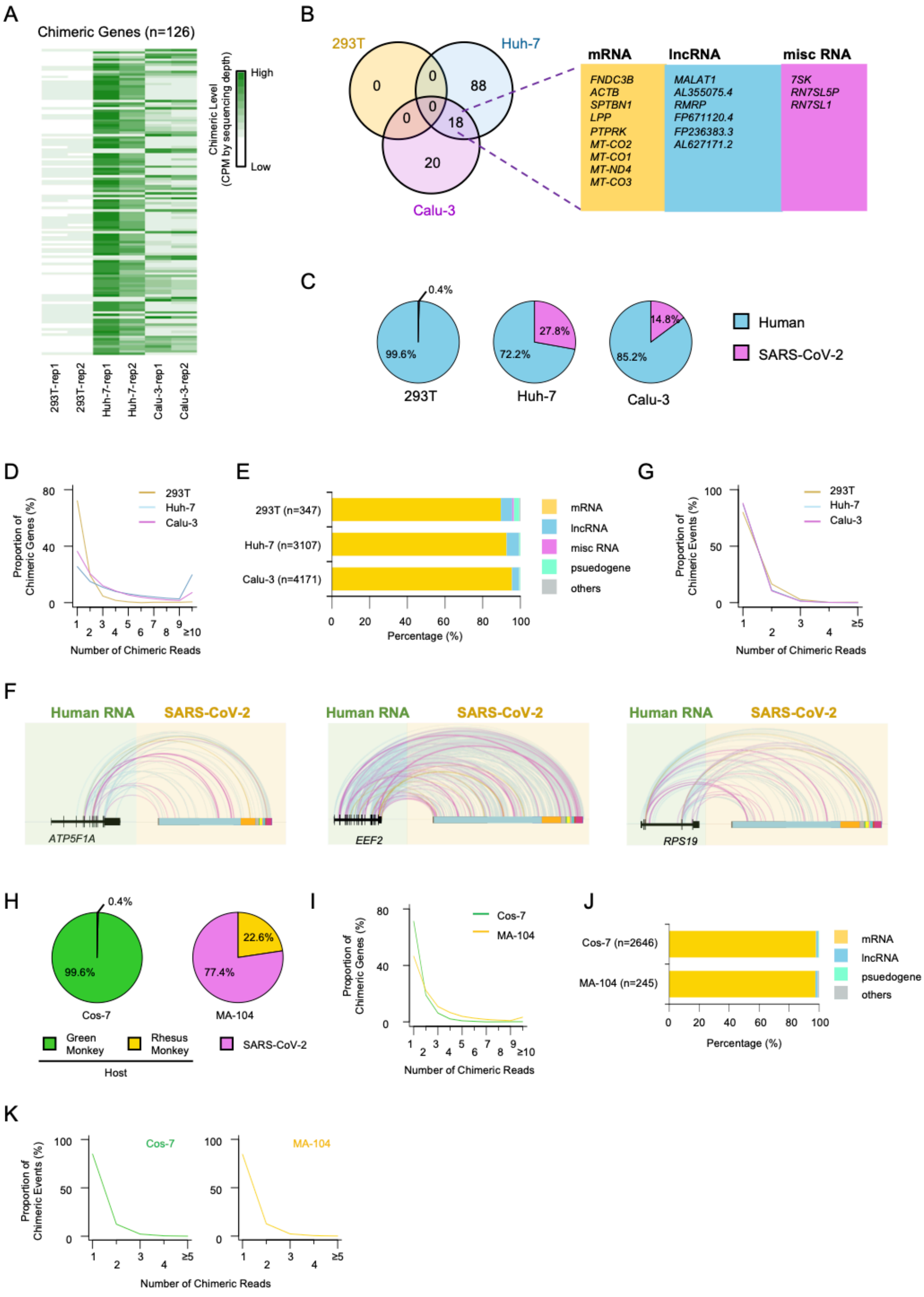

Figure S1. Viral chimeric gene transcripts were integrated with both mRNAs and non-coding RNAs from host.

(A) Heatmap displaying the chimeric level for filtered chimeric genes in each sample. The chimeric level was defined as CPM of chimeric reads and normalized by sequencing depth.

(B) Venn diagram showing the number of shared and specific chimeric genes from **(A)** among 3 human cell lines. The overlapped genes between Huh-7 and Calu-3 cells mainly encode mRNAs, lncRNAs and miscRNAs.
(C) Pie charts showing the proportions of reads aligned to human (blue) and SARS-CoV-2 (pink) genomes in infected 293T, Huh-7 and Calu-3 cells.
(D) Line chart displaying the proportion of chimeric genes supported by various numbers of chimeric reads in 293T (yellow), Huh-7 (blue) and Calu-3 (pink) cells.
(E) Barplot displaying the proportions of RNA types for chimeric genes in 3 human cell lines.
(F) IGV tracks displaying the junction loci in both human RNA and SARS-CoV-2 RNA for common chimeric gens *ATP5F1A* (top), *EEF2* (middle) and *RPS19* (bottom). The lines with different colors indicate the sources of chimeric reads. Yellow, blue and pink represents 293T, Huh-7 and Calu-3 cells, respectively. Blocks with different colors represent 5’UTR, ORF1ab, S, ORF3a, E, M, ORF6, ORF7ab, ORF8, N and 3’UTR along the SARS-CoV-2 genome from left to right.
(G) Line chart displaying the proportions of chimeric events supported by various numbers of chimeric reads in 293T (yellow), Huh-7 (blue) and Calu-3 (pink) cells.
(H) Pie charts showing the proportions of reads aligned to green monkey (green) and SARS-CoV-2 (pink) in infected Cos-7 cells (left), and proportions of reads aligned to rhesus monkey (yellow) and SARS-CoV-2 (pink) in infected MA-104 cells (right).
(I) Line chart displaying the proportion of chimeric genes represented by various numbers of chimeric reads in Cos-7 (green) and MA-104 (yellow) cells.
(J) Barplot displaying the proportion of RNA types for chimeric genes in Cos-7 and MA-104 cells.
(K) Line chart displaying the proportions of chimeric events represented by various numbers of chimeric reads in Cos-7 (left) and MA-104 (right) cells.

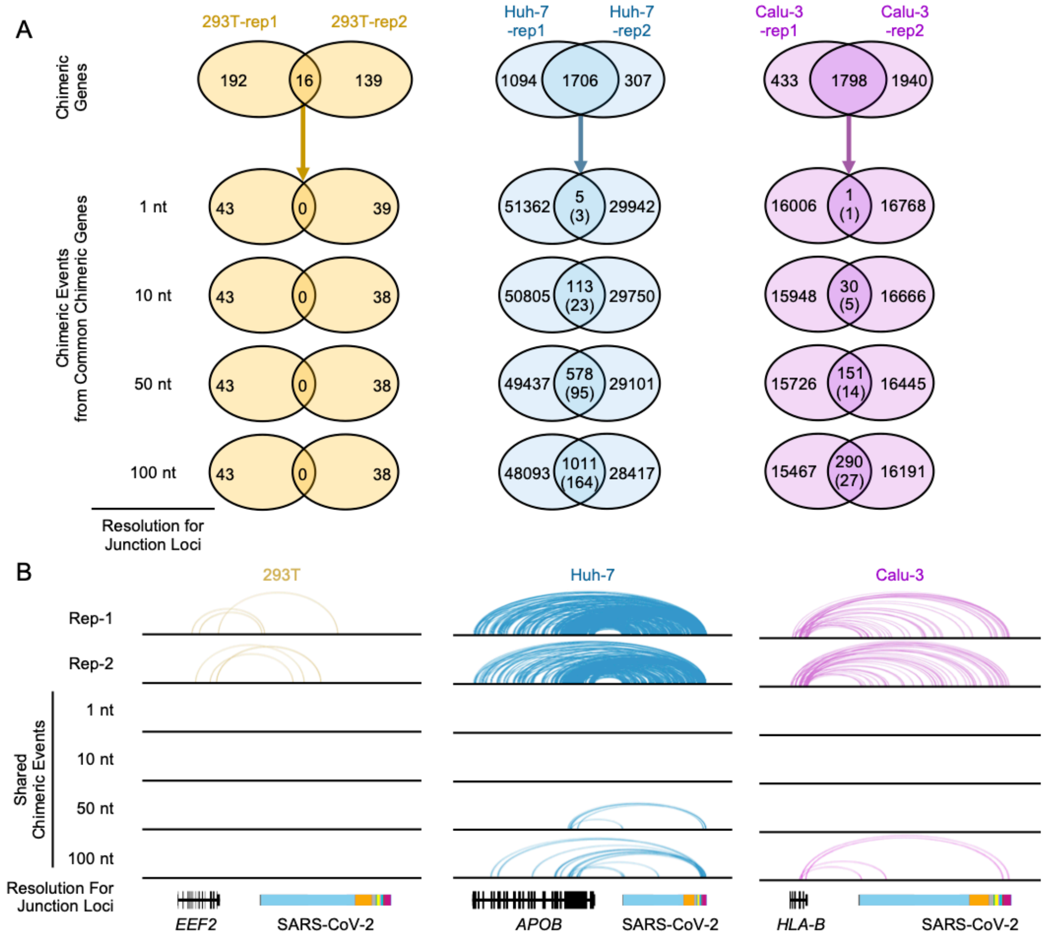

Figure S2. The chimeric events were not conserved between replicates.

(A) Venn diagrams showing the number of shared chimeric genes for two replicates from same cell line (top) and conserved chimeric events on shared chimeric genes at different resolutions (1 nt, 10 nt, 50 nt and 100 nt). For venn diagrams of chimeric events, the values in brackets represents the numbers of chimeric genes containing the shared chimeric events.

(B) IGV tracks displaying the junction loci in both human RNA and SARS-CoV-2 RNA for chimeric genes in each replicate (top). The shared chimeric events were displayed at different nucleotide resolutions (1 nt, 10 nt, 50 nt and 100 nt). Blocks with different colors represent 5’UTR, ORF1ab, S, ORF3a, E, M, ORF6, ORF7ab, ORF8, N and 3’UTR along the SARS-CoV-2 genome.

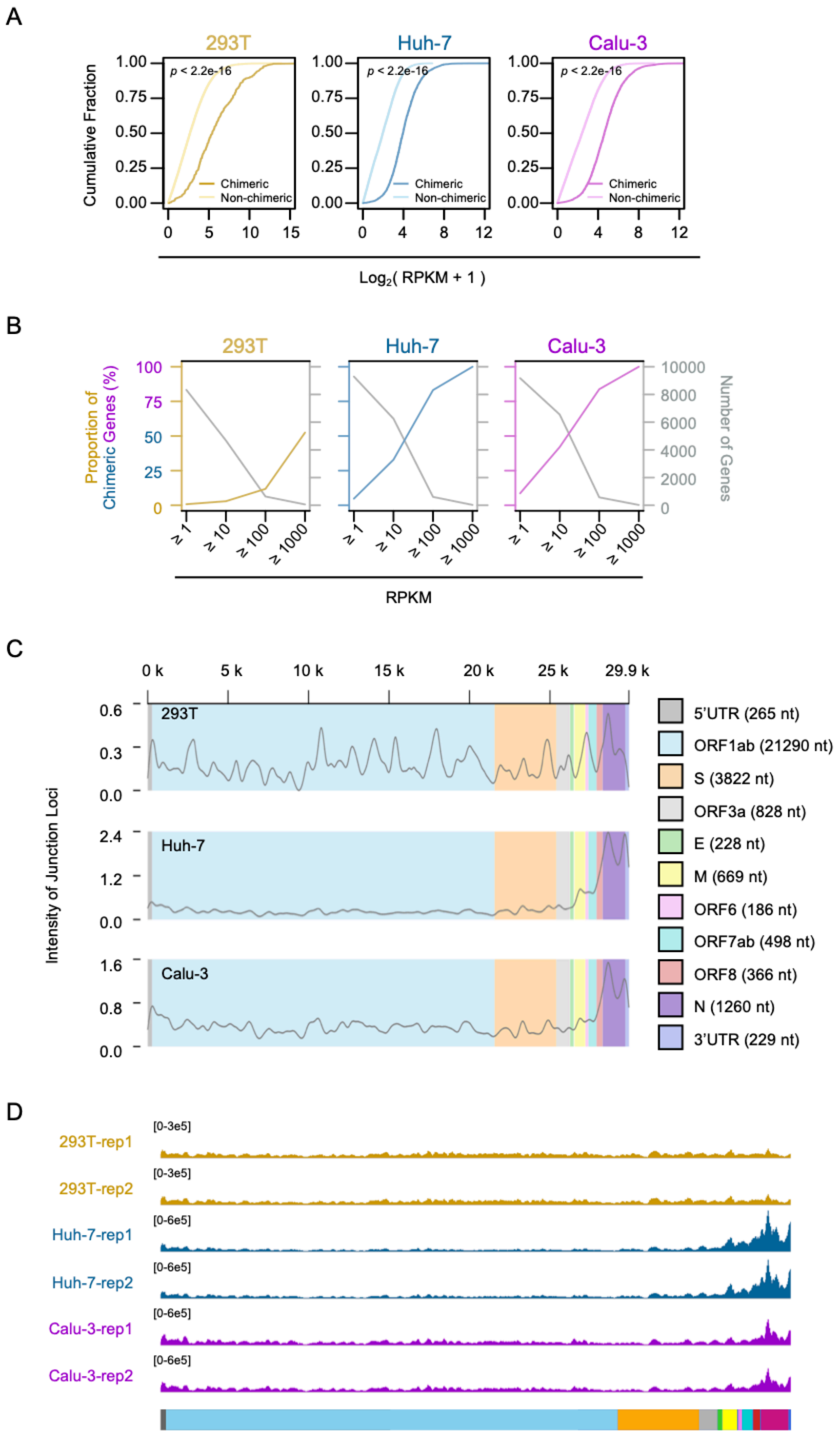

Figure S3. Chimeric genes were enriched in highly expressed genes for human and SARS-CoV-2.

(A) Cumulative distributions of expression levels for chimeric genes (dark color) and non-chimeric gens (light color) in 293T, Huh-7 and Calu-3 cells. *P* values were calculated by Mann-Whitney U test.

(B) Number of genes within different expression levels (grey) and proportion of

chimeric genes in each pool within various expression levels. Yellow, blue and pink represent 293T, Huh-7 and Calu-3 cells, respectively.

(C) IGV tracks displaying the intensity of chimeric reads along SARS-CoV-2 genome in infected 293T (top), Huh-7 (middle) and Calu-3 (bottom) cells.

(D) IGV tracks displaying the depth of perfect matched reads along SARS-CoV-2 genome in two replicates of each cell line.

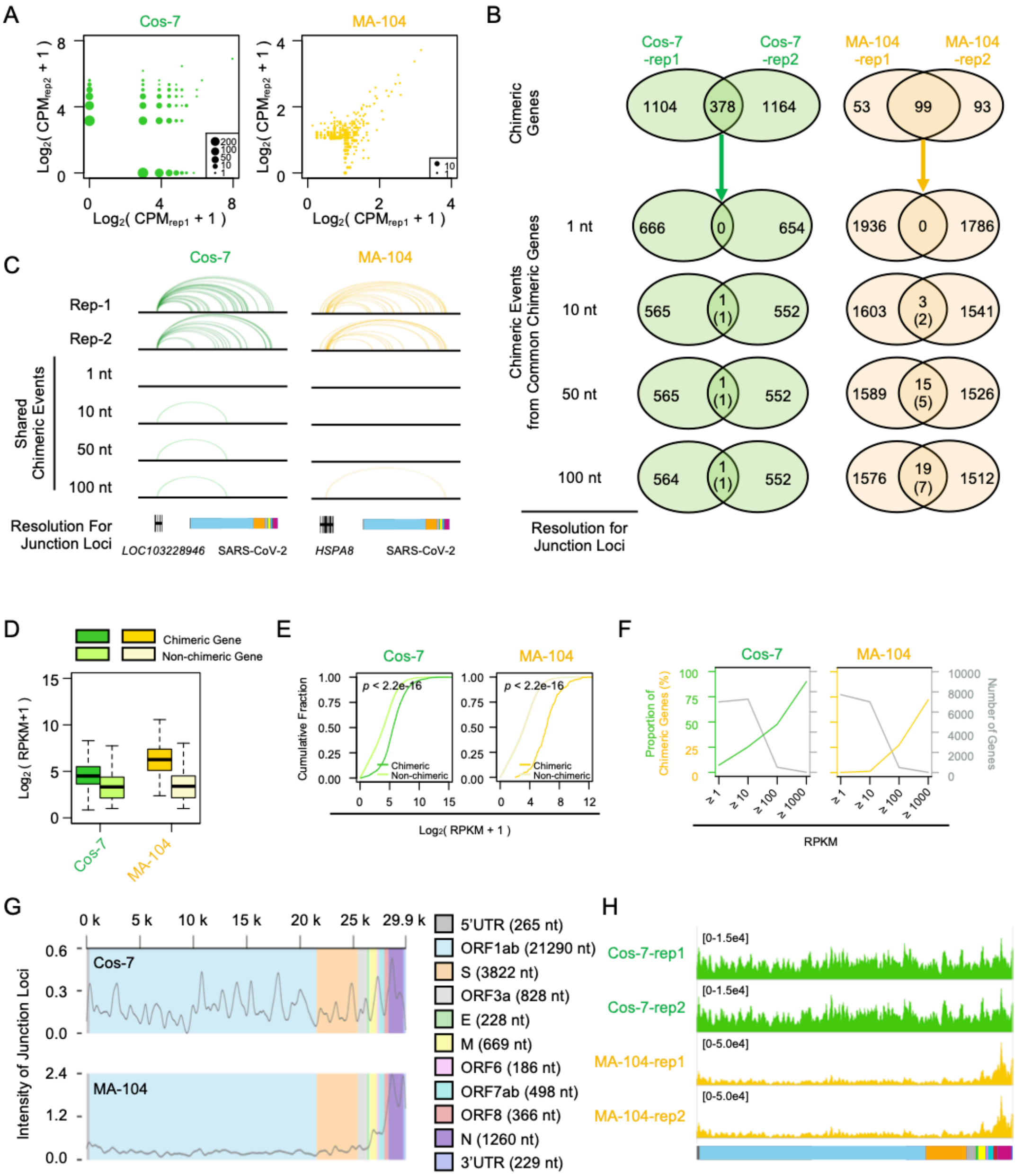

Figure S4. Both chimeric genes and events in Cos-7 (green monkey) and MA-104 (rhesus monkey) cells showed similar pattern to human.

(A) Scatter plot displaying the chimeric levels for chimeric genes in two replicates. The sizes of scatters represent number of chimeric genes with the same distribution of chimeric levels in two replicates.

(B) Venn diagrams showing the number of shared chimeric genes for two replicates of each cell line (top) and conserved chimeric events on shared chimeric genes at different nucleotide resolutions (1 nt, 10 nt, 50 nt and 100 nt). For venn diagrams of chimeric events, the values in brackets represent the numbers of chimeric genes containing the

shared chimeric events.

(C) IGV tracks displaying the junction loci in both monkey RNA and SARS-CoV-2 RNA for chimeric genes in two replicates (top). The shared chimeric events were displayed at different resolutions (1nt, 10nt, 50nt and 100nt). Blocks with different colors represent 5'UTR, ORF1ab, S, ORF3a, E, M, ORF6, ORF7ab, ORF8, N and 3'UTR along the SARS-CoV-2 genome.

(D) Boxplot displaying the distributions of expression level for chimeric genes (dark color) and non-chimeric genes (light color) in infected Cos-7 and MA-104 cells.

(E) Cumulative distributions of expression levels for chimeric genes (dark color) and non-chimeric gens (light color) in Cos-7 and MA-104 cells. *p* values were calculated by Mann-Whitney U test.

(F) Number of genes within different expression levels (grey) and proportion of chimeric genes in each pool within varicose expression levels.

(G) IGV tracks displaying the intensity of chimeric reads along SARS-CoV-2 genome in two replicates of infected Cos-7 (top) and MA-104 (bottom) cells.

(H) IGV tracks displaying the depth of perfect matched reads along SARS-CoV-2 genome.

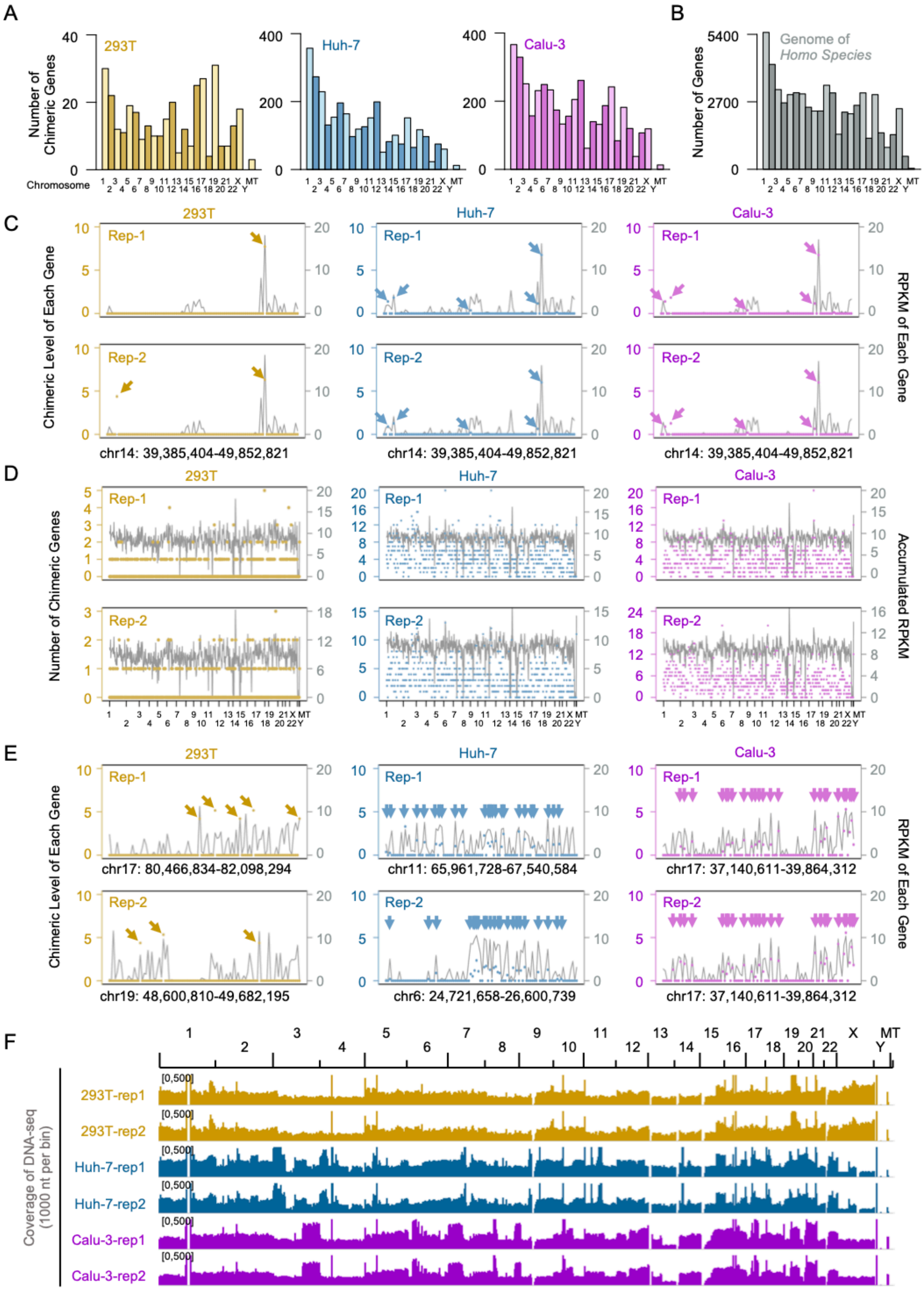

Figure S5. Information of chimeric genes along chromosomes and coverage of reads from whole genome sequencing.

(A) Barplot displaying the numbers of chimeric genes along different chromosomes in infected 293T, Huh-7 and Calu-3 cells.

(B) Barplot displaying the numbers of all annotated genes along different chromosomes.
(C) Chimeric levels and corresponding expression levels of genes along the bin with the highest accumulated chimeric levels (chr14: 39,385,404-49,852,821) were displayed by scatters and lines, respectively. The arrows point out the chimeric levels and gene loci of chimeric genes.
(D) The frequency and accumulated expression levels of chimeric genes in corresponding bins were displayed by scatters and lines, respectively.
(E) Chimeric levels and corresponding expression levels of genes along the bins with the most chimeric genes were displayed by scatters and lines, respectively. The arrows indicate the loci of chimeric genes.
(F) IGV tracks showing the coverage of whole genome sequencing along genome in each sample.

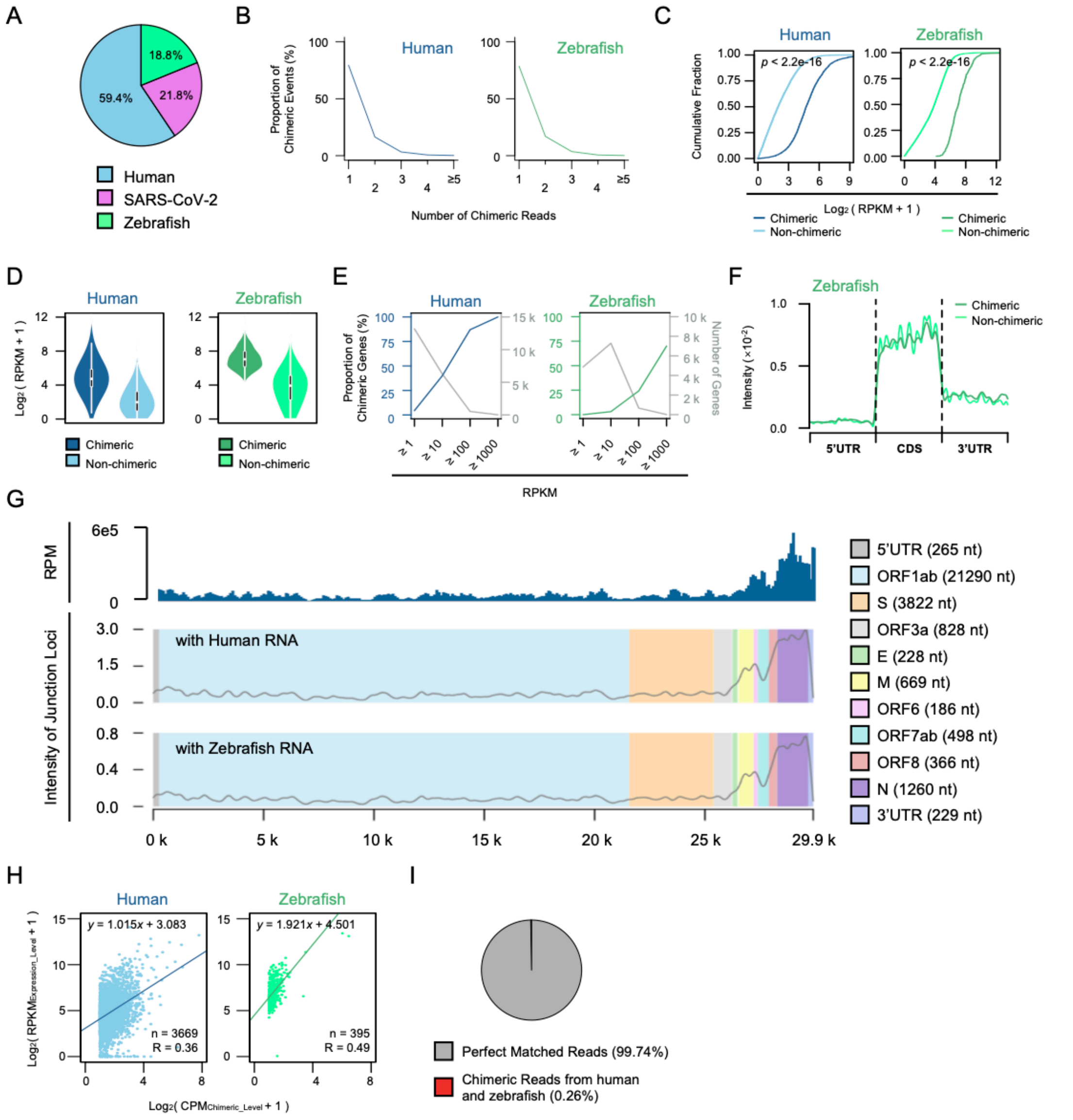

Figure S6. Chimeric events might come from library constructions but not natural events.

(A) Pie chart showing the proportions of reads aligned to human (blue), SARS-CoV-2 (pink) and zebrafish (green) in the mixed library.

(B) Line chart displaying the proportion of chimeric events represented by various numbers of chimeric reads in mixed library. Blue and green lines represent viral chimeric genes with human or zebrafish RNA, respectively.

(C) Cumulative distributions of expression levels for chimeric genes (dark color) and non-chimeric gens (light color) for human (left) and zebrafish (right) RNAs.

(D) Violin plot displaying the distributions of expression level for chimeric genes (dark color) and non-chimeric genes (light color) for human RNA (left) and zebrafish RNA

(right).

(E) Number of genes within different expression levels (grey) and proportion of chimeric genes in each pool within various expression levels. The left and right panels showing the human RNA and zebrafish RNA from the same mixed library, respectively.

(F) Distribution of chimeric (dark green) and non-chimeric reads (light green) across the length of chimeric zebrafish mRNAs. 5'UTRs, CDSs, and 3'UTRs of human mRNAs were individually binned into regions spanning 1% of their total length, and the percentages of chimeric and non-chimeric reads that fall within each bin were determined, respectively.

(G) IGV tracks displaying the depth of viral RNA along SARS-CoV-2 genome (top) and intensity of chimeric reads with human and zebrafish, individually (bottom).

(H) Scatter plot displaying the correlation between chimeric level and expression level (RPKM) of chimeric genes for human (left) and zebrafish (right) from the same mixed library.

(I) Pie chart showing the proportions of reads identified as perfect matched reads (including reads aligned to human, zebrafish and SARS-CoV-2), and human-zebrafish chimeric reads (red).
